# HIV Risk Perception, Sexual Behavior, and HIV Prevalence among Men-Who-Have-Sex-with-Men at a Community-Based Voluntary Counseling and Testing Center in Kuala Lumpur, Malaysia

**DOI:** 10.1155/2014/236240

**Published:** 2014-06-25

**Authors:** Kwee Choy Koh, Lit Sin Yong

**Affiliations:** ^1^Department of Medicine, Clinical School, International Medical University, Jalan Rasah, 70300 Seremban, Negeri Sembilan, Malaysia; ^2^Department of Medicine, Hospital Tuanku Ja'afar Seremban, Jalan Rasah, 70300 Seremban, Negeri Sembilan, Malaysia

## Abstract

We describe the HIV risk perception, sexual behavior, and HIV prevalence among 423 men-who-have-sex-with-men (MSM) clients who received voluntary counseling and testing (VCT) services at a community-based center in Kuala Lumpur, Malaysia. The mean age was 29 years old. One hundred one (23.9%) clients rated themselves as low risk, 118 (27.9%) as medium risk, 36 (8.5%) as high risk, and 168 (39.7%) were unsure of their risk. Twenty-four (9.4%) clients tested HIV positive (4 (4%) low risk, 9 (7.6%) medium risk, 11 (30.6%) high risk, and 13 (7.7%) unsure risk). We found a positive correlation between risk perception and HIV infection in this study. Clients with high HIV risk perception have 17x the odds of testing HIV positive compared to low risk clients. High HIV risk perception was significantly associated with multiple sex partners, multiple types of sex partners, alcohol use before intercourse, unprotected sex beyond 6 months, and inconsistent condom use during anal sex compared to low risk clients. There were no statistically significant differences between medium risk and unsure risk clients compared to low risk clients. Strategies should be targeted towards change in sexual practices among those who are perceived to be at high risk.

## 1. Introduction

HIV risk perception studies among men-who-have-sex-with-men (MSM) have often reported similar findings, namely: inaccurate or misperception of HIV risk being common among MSM [1, 2], perception of low risk being associated with no previous HIV testing and considerable high risk behavior [3–5], and moderate/high risk being associated with unrecognized HIV infection [4].

Compared to the general adult population, MSM in Asia have 18.7 times the odds of being HIV infected [6]. Across Asia, HIV prevention expenditures targeting MSM have been reported to range between 0% in China to only 4% in Thailand [7]. The lack of funding for these programs is lamentable as prevention and harm reduction strategies targeting MSM are successful in reducing high-risk behaviors [8, 9]. In Malaysia, homosexuality is illegal and stigmatization is a problem. MSM in Malaysia are part of the marginalized most-at-risk populations (MARP) that include commercial sex workers, migrant workers, and transgender people. HIV prevention programs including HIV testing among the MARP in Malaysia are mainly spearheaded by several nongovernmental organizations (NGO) with very limited resources. We present the findings of a study where we explored the relationship between HIV risk perception in a cohort of MSM who sought VCT services at a NGO-run center in Kuala Lumpur, Malaysia, and their sexual behavior and risk factors. Our study shows that there exists a significant association between high HIV risk perception with high risk sexual behavior and HIV positivity. Targeting MSM with high risk perception may indeed be the strategy to consider in a resource-limited setting in order to gain maximum benefits among the MSM in Malaysia.

## 2. Materials and Methods

Data were collated from the pre-HIV testing questionnaires filled by clients seeking VCT services at a community-based VCT centre from January 2008 to December 2008. Completion of the questionnaire was voluntary and no data identifiable to the client were required. A total of 740 clients sought VCT services at the centre between January 1, 2008, and December 31, 2008. Of these, 433 clients disclosed their sexuality as either homosexual or bisexual and were collectively categorized as MSM in this study. The profiles, sexual practices, and HIV prevalence of these 433 clients have been reported in earlier studies [10, 11].

The clients were asked to rate their own perceived HIV risk in the questionnaire. Risk perception was stratified into four groups: “low risk” (<25% chance), “medium risk” (25–75% chance), “high risk” (>75% chance), and “unsure risk.” Information regarding the clients' sexual behaviors, preferred role during anal sex, and other risk factors such as multiple sexual partners, types of sexual partners, alcohol use before sex, history of sexually transmitted infections (STIs), last unprotected sex, and condom use during anal sex, all in the preceding six months, was collated. Rapid test for HIV infection was performed using either the SD Bioline HIV test (Standard Diagnostics Inc.) or the ACON HIV test kits according to the manufacturer's specification.

Descriptive analysis was used for description of the demographic characteristics, sexual behaviors, and risk factors for acquisition of HIV infection. Chi-square analysis was used to determine the odds ratio of HIV acquisition in each of the risk groups. Regressions analysis was used to determine which of the risk factors were predictors of HIV acquisition in the low, medium, high, and unsure risk perceptions groups. All statistical analyses were performed using SPSS for Windows v21. A *P* value of <0.05 with 95% confidence interval was deemed significant. This study received approval from the National Medical Research Register of Malaysia.

## 3. Results 

### 3.1. Demographic Characteristics

Four hundred twenty-three clients rated their own HIV risk perception in this study of which 350 (82.7%) identified themselves as homosexuals while 73 (17.3%) as bisexuals. The mean age was 29.2 years old. The youngest client was 18 years old and the oldest was 61 years old. Most of the clients were Chinese (*N* = 250) followed by Malays (*N* = 115). Most had received tertiary education (*N* = 263) and earned a monthly income between MYR 2001 and 5000 (*N* = 152). The details of the demographic parameters are shown in Table 1.

Two hundred fifty-six clients (60.5%) reported receiving VCT services at the same center previously prior to the survey. The breakdown according to risk categories was: low risk—64.4%, medium risk—67.8%, high risk—77.8%, and unsure risk—49.4%, respectively.

### 3.2. HIV Risk Perception

From a total of 423 clients, 101 (23.9%) clients perceived themselves to be at low risk (<25%) of being infected with HIV at the time of survey, 118 (27.9%) perceived themselves to be at medium risk (25–75%), 36 (8.5%) clients perceived themselves to be at high risk (>75%), while the remaining 168 (39.7%) clients were unsure regarding their risk.

### 3.3. Sexual Behavior and Preferred Role during Sex

Four hundred fifteen clients disclosed their sexual behaviors in the preceding 6 months. Most had engaged in anal and oral sex (*N* = 328, 79.0%), 25 (6.0%) had vaginal, anal, and oral sex, 23 (5.5%) had anal sex only, 22 (5.3%) had oral sex only, 9 (2.2%) had vaginal and oral sex only, 6 (1.4%) had vaginal sex only, and 2 (0.5%) had vaginal and anal sex only (Table 1). Out of 287 clients who disclosed their preferred sexual role during anal sex, 140 (48.8%) were “versatile,” 72 (25.1%) were “top,” and 75 (26.1%) were “bottom” (Table 1).

### 3.4. Other Risk Factors for HIV Acquisition

Table 2 shows the number of clients in each risk perception group who had exposure to risk factors for HIV transmission in the preceding 6 months. The risk factors were multiple sexual partners (10 or less versus >10), promiscuity (regular versus regular and casual/transactional sex partners), alcohol use before sex, history of STIs, last unprotected sex, and condom use during anal sex.

An item in the questionnaire explored drug use before or during sex in the preceding 6 months. Out of 411 clients who responded to this question, only 42 (10.2%) clients reported drug use before or during sex. As the number of respondents was small, meaningful conclusions using statistical methods could not be made.

### 3.5. Odds Ratio

Assigning clients who rated themselves to be at “low risk” to acquire HIV infection as the reference group, we determined the odds ratio for testing HIV positive in each of the other risk groups (i.e., medium risk, high risk, and unsure risk) for each of the risk factors for HIV transmission. The results are shown in Table 3.

The odds of testing positive for HIV infection in clients who rated themselves to be at “high risk” were 17x higher compared to clients in the “low risk” group (OR 17.14, 95% CI: 3.28–89.72, *P* < 0.001) while clients who rated themselves to be at “medium risk” had 2.6x the odds of testing positive for HIV compared to “low risk” clients (OR 2.61, 95% CI: 0.51–13.41, *P* = 0.251). Clients who were unsure of their risk had 1.9x the odds of testing HIV positive compared to “low risk” clients (OR 1.96, 95% CI: 0.40–9.75, *P* = 0.410). The increased odds in the “medium risk” and “unsure risk” groups were not statistically significant compared to the “low risk” group.

Risk factors which were significant predictors for HIV acquisition in the “high risk” clients include having more than 10 sexual partners in the preceding 6 months, having casual and/or transactional sex partners in the preceding 6 months, alcohol use before sex in the preceding 6 months, unprotected sex of more than 6 months ago, and inconsistent condom use during anal sex in the preceding 6 months (all *P* < 0.05). The same factors were not statistically significant predictors in the “medium risk” and “unsure risk” groups (*P* > 0.05). History of sexually transmitted infections was not a predictor for HIV acquisition in any of the risk groups in this study (Table 3).

## 4. Discussion

MSM who rated themselves to be at high risk for being infected with HIV in this study were significantly associated with high risk factors and were 17x more likely to be infected with HIV compared to those who rated themselves to be at low risk. The significant predictors of HIV transmission in the high HIV risk perception group were multiple sex partners, promiscuity, alcohol use before sex, unprotected sexual intercourse, and inconsistent condom use during anal sex (Tables 2 and 3). In contrast, clients who have rated themselves to be at medium risk or were unsure of their risk were not significantly associated with these risk factors and had lower odds of being infected with HIV compared to clients who rated themselves as “low risk.” These risk factors have been well documented to be independent risk factors for HIV transmission among MSM [12–14].

The findings suggest that the clients were aware of the* degree* of their own high risk behaviors which enabled them to properly rate themselves into each of the HIV risk perception categories. This is in contrast to other studies among MSM that have shown that misperception of risk is the usual finding [1, 3–5]. About 60% of the clients in our study had previously used the VCT services at the same center prior to the survey. These clients would most likely have received the prerequisite pre- and posttest counseling previously which may have contributed to greater awareness of their own risk factors. This may account for the clients' ability to rate their perceived risk fairly accurately.

Nevertheless, clients who did not rate themselves to be at “high risk” of HIV infection also reported considerable risk behaviors with similar proportions. The only exception was that of inconsistent condom use during anal sex with 91.4% of clients in the high HIV risk group compared to 67.9%, 75.2%, and 79% in the low, medium, and unsure risk groups, respectively (Table 2).

Surprisingly, history of STI was not a significant predictor for HIV transmission in any of the risk perception groups in our study. We believe this may be due to the structure and the anonymous nature of the questionnaire used in our study where detailed information regarding the types of STI and therapy received could not be readily obtained nor verified. Most clients were disinclined to reveal more information regarding the STI which they have had. Similarly, drug use is a legal offense in Malaysia and is punishable by law with either prolonged incarceration or capital punishment. This may have led to the reluctance of the clients to honestly disclose history of drug use before or during sexual intercourse making it difficult to interpret the available data meaningfully.

We recognized several major themes that have emerged from the findings in our study, namely, (1) MSM in this study were able to accurately rate their perceived risk of HIV infection which suggests they were aware of their individual risk factors; (2) the heightened awareness of the risk factors may have been contributed in part by previous counseling sessions these clients have received; (3) MSM in the high HIV risk perception group were associated with significant risk predictors of HIV transmission and were at much higher odds testing HIV positive compared to clients in the “low risk” category; and (4) despite rating themselves into lower risk groups, the MSM in these groups reported considerable risk behaviors suggesting the problem of misperceptions does exist to a certain degree in this study population.

This study has limited generalizability because it was confined to MSM who utilized the VCT services in one center and, therefore, may not be applicable to MSM who do not use VCT services. Nevertheless, in the setting of limited funding and resources, the findings in this study suggest that focused strategies to educate MSM regarding HIV and STI risks, especially in the high HIV risk perception group and efforts to raise personal risk perceptions, are critical because self-perception often guides the individual's testing and preventive behaviors [2]. Because some of the clients who reported lower risk perceptions in this study were found to be HIV-infected; consistent condom use during sexual intercourse regardless of the self-perceived risk with or without other risk factors should be the primary message to MSM during every VCT encounter.

In conclusion, MSM in this study could rate their HIV risk with reasonable accuracy. Perceived high HIV risk was associated with many of the established risk factors for transmission of HIV and STI. High risk perception was positively correlated with HIV infection. Strategies designed to change the sexual practices among those who perceived themselves to be at high risk of being infected with HIV should be put in place in a limited-resource setting.

## Figures and Tables

**Table 1:** Demographic characteristics and sexual behavior of MSM clients.

Demography and sexual behavior	Perceived risk			
	Low	Medium	High	Unsure
(1) Age group in years (*N* = 421)∗				
<20	3 (42.9%)	0	0	4 (57.1%)
20–29	50 (19.7%)	78 (30.7%)	23 (9.1%)	103 (40.6%)
30–39	29 (22.8%)	33 (26.0%)	12 (9.4%)	53 (41.7%)
40–49	11 (44.0%)	6 (24.0%)	1 (4.0%)	7 (28.0%)
50–59	4 (66.7%)	1 (16.7%)	0	1 (16.7%)
>60	2 (100%)	0	0	0
(2) Ethnic group (*N* = 419)∗				
Malay	15 (13.0%)	30 (26.1%)	11 (9.6%)	59 (51.3%)
Chinese	64 (25.6%)	75 (30.0%)	20 (8.0%)	91 (36.4%)
Indian	6 (31.6%)	3 (15.8%)	0	10 (52.6%)
Other	15 (42.9%)	10 (28.6%)	3 (8.6%)	7 (20.0%)
(3) Sexuality (*N* = 423)∗				
Homosexual	82 (23.4%)	101 (28.9%)	33 (9.4%)	134 (38.3%)
Bisexual	19 (26.0%)	18 (23.3%)	3 (4.1%)	34 (46.6%)
(4) Education level (*N* = 353)∗				
Primary	4 (25.0%)	2 (12.5%)	0	10 (62.5%)
Secondary	19 (25.7%)	23 (31.1%)	5 (6.8%)	27 (36.5%)
Tertiary	67 (25.5%)	78 (28.7%)	23 (8.7%)	95 (36.1%)
(5) Monthly income (MYR) (*N* = 394)∗				
<1000	6 (20.0%)	9 (30.0%)	4 (13.3%)	11 (36.7%)
1000–2000	15 (16.9%)	27 (30.3%)	10 (11.2%)	37 (41.6%)
2001–5000	37 (24.3%)	44 (28.9%)	16 (10.5%)	55 (36.2%)
>5000	16 (39.0%)	11 (26.8%)	3 (7.3%)	11 (26.8%)
(6) Sexual preference (*N* = 415)∗				
Anal and oral	71 (21.6%)	98 (28.8%)	32 (9.7%)	128 (38.9%)
Oral only	9 (40.9%)	5 (22.7%)	0	8 (36.4%)
Vaginal and anal and oral	6 (24.0%)	6 (24.0%)	1 (4.0%)	12 (48.0%)
Anal only	6 (26.1%)	4 (17.4%)	2 (8.7%)	11 (47.8%)
Vaginal only	4 (66.7%)	1 (16.7%)	0	1 (16.7%)
Vaginal and oral	3 (33.3%)	1 (11.1%)	0	5 (55.5%)
Vaginal and anal	0	2 (100%)	0	0
(7) Role during anal sex (*N* = 287)∗				
Bottom	11 (14.7%)	18 (24.0%)	11 (14.7%)	35 (46.7%)
Top	21 (29.2%)	21 (29.2%)	3 (4.2%)	27 (37.3%)
Versatile	32 (22.9%)	42 (30.0%)	11 (7.9%)	55 (38.3%)

*Number of clients who responded to this part of the pre-HIV testing questionnaire.

MYR: Malaysian ringgit.

**Table 2:** Risk factors and prevalence of HIV in each perceived risk group.

Risk factors (preceding 6 months)	Perceived risk			
	Low	Medium	High	Unsure
(1) Number of sex partners (*N* = 273)∗				
≤10	34 (54.8%)	46 (61.3%)	12 (54.5%)	64 (56.1%)
>10	28 (45.2%)	29 (39.7%)	10 (45.5%)	50 (43.9%)
(2) Types of sex partners (*N* = 398)∗				
Regular	44 (46.3%)	33 (28.9%)	13 (40.6%)	66 (42.0%)
Regular and casual/transactional sex	51 (53.6%)	81 (71.1%)	19 (59.4%)	91 (58.0%)
(3) Alcohol use before sex (*N* = 313)∗				
No	31 (40.8%)	37 (44.6%)	14 (53.8%)	72 (56.3%)
Yes	45 (59.2%)	46 (55.4%)	12 (46.2%)	56 (43.4%)
(4) History of sexually transmitted infections (*N* = 412)∗				
No	74 (75.5%)	91 (79.1%)	24 (68.6%)	137 (83.5%)
Yes	24 (24.5%)	24 (20.9%)	11 (31.4%)	27 (16.5%)
(5) Last unprotected sex (*N* = 423)∗				
<6 months ago	49 (48.5%)	82 (69.5%)	27 (75.0%)	94 (56.0%)
>6 months ago	52 (51.5%)	36 (30.5%)	9 (25.0%)	74 (44.0%)
(6) Condom use during anal sex (*N* = 363)∗				
Consistent	25 (32.1%)	22 (21.0%)	3 (8.6%)	36 (24.8%)
Inconsistent	53 (67.9%)	83 (79.0%)	32 (91.4%)	109 (75.2%)
(7) HIV result (*N* = 423)∗				
Negative	97 (96.0%)	109 (92.4%)	25 (69.4%)	155 (92.3%)
Positive	4 (4.0%)	9 (7.6%)	11 (30.6%)	13 (7.7%)

*Number of clients who responded to this part of the pre-HIV testing questionnaire.

**Table 3:** Odds of testing HIV positive in each perceived risk group for each of the risk factors using “low risk” as reference.

Risk factor	Odds ratio	*P* value	95% CI∗
(1) >10 Sex partners in preceding 6 months			
Medium risk	1.364	0.761	0.18–10.06
High Risk	42.00	<0.001	8.83–258.47
Unsure risk	1.475	0.676	0.24–9.15
(2) Promiscuity (had > than regular sex partner) in preceding 6 months			
Medium risk	2.002	0.142	0.60–6.71
High Risk	10.670	0.019	3.13–36. 36
Unsure risk	2.034	0.465	0.65–6.42
(3) Alcohol use before sex in preceding 6 months			
Medium risk	2.002	0.613	0.60–6.71
High Risk	10.670	<0.001	3.13–36.36
Unsure risk	0.041	0.928	0.65–6.42
(4) History of sexually transmitted infections			
Medium risk	1.995	0.156	0.60–6.69
High Risk	10.771	0.261	3.15–36.81
Unsure risk	1.855	0.800	0.60–5.92
(5) Last unprotected sex >6 months ago			
Medium risk	2.002	0.185	0.60–6.71
High Risk	10.670	<0.001	3.12–36.36
Unsure risk	2.034	0.505	0.65–6.42
(6) Inconsistent condom use during anal sex in preceding 6 months			
Medium risk	3.259	0.527	1.05–10.09
High Risk	12.182	<0.001	3.80–39.03
Unsure risk	2.412	0.712	0.78–7.42

*CI: confidence interval up to 2-decimal point.
